# Methods for monitoring cancer cell pyroptosis

**DOI:** 10.20892/j.issn.2095-3941.2021.0504

**Published:** 2021-12-22

**Authors:** Shuo Wang, Yuantong Liu, Lu Zhang, Zhijun Sun

**Affiliations:** 1 The State Key Laboratory Breeding Base of Basic Science of Stomatology (Hubei-MOST) & Key Laboratory of Oral Biomedicine, Ministry of Education, School and Hospital of Stomatology, Wuhan University, Wuhan 430079, China; 2 Department of Oral Maxillofacial-Head Neck Oncology, School and Hospital of Stomatology, Wuhan University, Wuhan 430079, China

**Keywords:** Pyroptosis, gasdermin, caspase, cancer immunotherapy, cell death

## Abstract

Pyroptosis is a form of proinflammatory cell death that depends on the gasdermin family of proteins. The main features of pyroptosis are altered membrane permeability, cell swelling, membrane rupture, and the ability to mobilize a strong immune response. The relationship between pyroptosis and cancer has become a popular topic in immunological research. Multiple strategies for inducing pyroptosis in cancer cells have been developed for cancer therapy, including chemotherapy, small molecule drugs, and nanomedicines. In this review, we systematically discuss recent advances in research on the mechanisms of pyroptosis, and compare pyroptosis with apoptosis and necroptosis from several aspects. The development of various experimental systems has accompanied rapid progress in this field, but little consensus on monitoring pyroptosis is currently available. We focus on techniques commonly used to monitor pyroptosis, and describe future techniques that may be used to increase our knowledge in this field. Overall, the advancement of pyroptosis detection methods will help researchers to better investigate the relationships between pyroptosis and various cancers, and should provide insights into the use of these promising tools for cancer treatments.

## Introduction

In recent years, immunotherapy for tumors based on immune checkpoint inhibitors (ICIs) has revolutionized the field of cancer treatment^1,2^. Although ICIs have shown outstanding clinical efficacies, the number of patients benefiting from immunotherapy is limited^3–6^. An important reason is that killing cancer cells through the induction of non-inflammatory apoptosis often appears to be “immunologically silenced,” and suppresses subsequent immune responses^7,8^. Considering the innate resistance of tumors to apoptosis, the induction of cell death through a pathway other than apoptosis has emerged as a new strategy for tumor treatment. Unlike apoptosis, pyroptosis is a pro-inflammatory form of cell death dependent on the gasdermin (GSDM) family of proteins^9^. Pyroptosis is manifested by the rapid rupture of cell membranes and the release of many inflammatory molecules, resulting in a robust immune response^9^.

**Figure 1:**
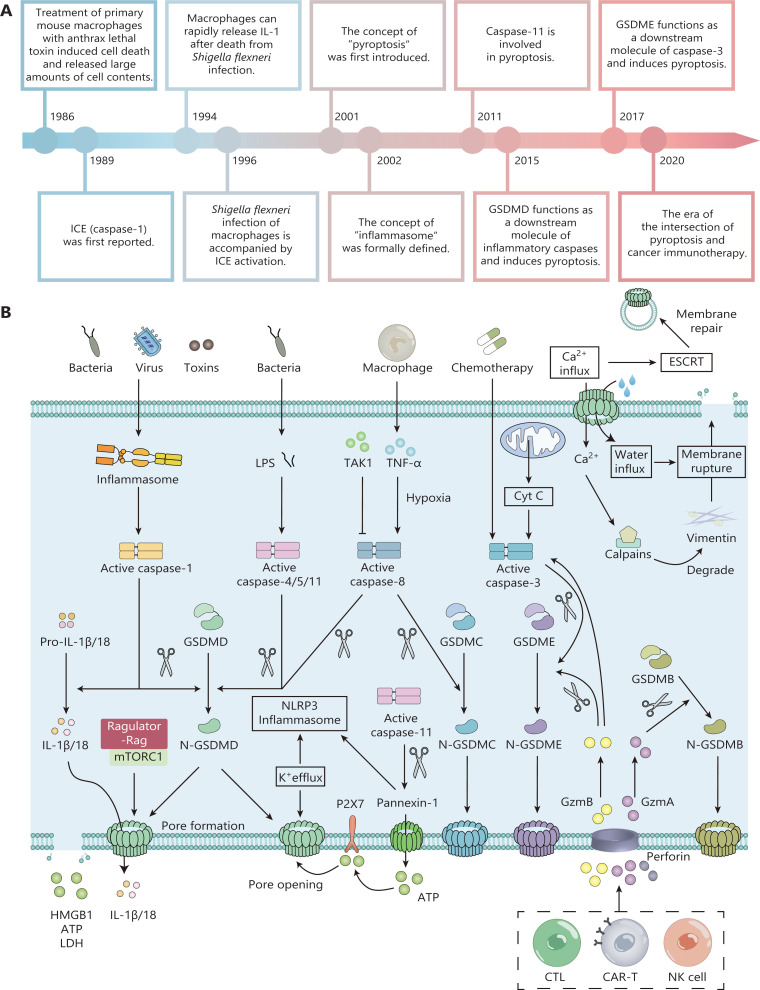
The timeline and molecular mechanisms of pyroptosis. (A) Critical events in pyroptosis research. (B) Molecular mechanisms of pyroptosis. In the canonical pathway, upon stimulation with bacteria, viruses, or toxins, pattern recognition receptors on the cell surface activate downstream signaling pathways. Activated caspase-1 specifically cleaves gasdermin D (GSDMD) to produce GSDMD-N termini. The cleaved GSDMD-N fragment disrupts cell membrane integrity and induces cellular pyroptosis. In addition, activated caspase-1 promotes the production of mature IL-1β and IL-18, which induce an inflammatory response. Caspases-4/5/11 mainly mediate the noncanonical pathway of pyroptosis. Upon stimulation with bacterial lipopolysaccharide, activated caspases-4/5/11 specifically cleave GSDMD, leading to the formation of GSDMD pores that cause K^+^ efflux, thereby activating the NLRP3-caspase-1 signaling pathway and amplifying the inflammatory response. In addition, the formation of GSDMD pores is accompanied by Ca^2+^ efflux, which activates the calpain mechanism that promotes membrane rupture and the endosomal sorting complex required for the transport mechanism that promotes membrane repair. Various chemotherapeutic agents induce tumor cell pyroptosis by inducing the caspase-3-GSDME axis. Inhibition of TGF-β-activated kinase 1 (TAK1) induces pyroptosis in mouse macrophages through the caspase-8-GSDMD pathway. In addition, during hypoxic conditions, TNF-α produced by macrophages induces cancer cell pyroptosis using the caspase-8-GSDMC pathway.

The earliest known description of pyroptosis was in 1986 (**Figure 1A**). Friedlander^10^ found that treating primary mouse macrophages with anthrax lethal toxin induced cell death and released large amounts of cellular contents. Subsequently, Brennan et al.^11^ identified a caspase-1-dependent cell death mode in *Salmonella*-infected macrophages with significant differences from conventional apoptosis. *Salmonella*-induced cell death was not accompanied by the activation of caspase-3 and PARP. In addition, the cell membranes of these dying cells were disrupted, yet apoptotic cells maintained their cell membrane integrity. Therefore, the concept of “pyroptosis” was first introduced in 2001 to describe this mode of cell death, which differed from apoptosis^12,13^. In 2011, Kayagaki et al.^14^ reported that some Gram-negative bacteria intracellularly activated caspase-11, which in turn caused cellular pyroptosis without relying on caspase-1. Subsequent studies confirmed that caspases-4/5/11 directly bound lipopolysaccharide (LPS) in the absence of a receptor (e.g., NLRP3) and directly induced pyroptosis without the action of caspase-1^15^. In 2015, Shi et al. reported that gasdermin D (GSDMD) was a downstream molecule of caspases-4/11, and was involved in inflammasome activation and pyroptosis^16–18^. This is another critical downstream substrate of inflammatory caspases identified after IL-1β. Subsequently, Aglietti et al. further elucidated the specific mechanism of pyroptosis caused by GSDMD^19–22^. Inflammatory caspases (e.g., caspases-1/4/5/11) specifically cleave GSDMD, producing 2 peptides, GSDMD-N and GSDMD-C. GSDMD-N binds to plasma membranes, causing cell membrane pore formation and inducing pyroptosis. Further studies confirmed that other members of the GSDM family, such as GSDME, had similar functions when hydrolyzed by caspase-3^23^. These findings finally identified the effector proteins of pyroptosis as members of the GSDM family.

Recent studies have revealed that cellular pyroptosis is closely related to the development and progression of cancer^24–26^. Because one of the characteristics of tumors is the evasion of apoptosis, the induction of pyroptosis is important in the anti-apoptotic treatment of tumors^8^. The induction of pyroptosis can overcome tumor resistance to apoptosis, induce sensitization to chemotherapy, and promote anti-tumor immune responses^24^. Pyroptosis of less than 15% of cancer cells is sufficient to clear the entire tumor, indicating the influential role of anti-tumor immunity in tumor killing that is induced by pyroptosis^27^. Multiple strategies for inducing pyroptosis in cancer cells have been developed for cancer therapy, including the use of chemotherapy, small molecule drugs, and nanomedicines^24^. However, the mechanism only of GSDMD-induced cell pyroptosis is presently clearly known. Therefore, further studies of the mechanisms of pyroptosis in different cancer cells and the proteins associated with the upstream and downstream signaling pathways involved should provide new tools for treating related tumors.

In this review, we systematically describe the molecular mechanisms of pyroptosis. As a newly discovered programmed death pathway, pyroptosis participates in extensive crosstalk with apoptosis and necroptosis. We therefore compared pyroptosis with apoptosis and necroptosis. As studies of pyroptosis have increased, new methods for monitoring cellular pyroptosis have emerged. Although many reviews have described the close relationship between pyroptosis mechanisms and various cancers, recent advances in pyroptosis detection lack systematic descriptions. An important and meaningful objective is therefore to identify various methods for monitoring pyroptosis. In this review, we focus on techniques commonly used to detect pyroptosis (including changes in cell morphology, staining status, molecular biomarkers, and *in vivo* assays), and provide descriptions of techniques that may be used in future studies. The identification of methods for monitoring pyroptosis will help researchers to better understand the relationships between pyroptosis and cancers, as well as provide references and new ideas for future studies.

## Molecular mechanisms of pyroptosis

The assembly of inflammasomes is the initial step in the canonical pathway of cellular pyroptosis. Inflammasomes are a class of receptors that recognize danger-associated intracellular molecular patterns (DAMPs) and pathogen-associated molecular patterns (PAMPs)^28^. For example, upon stimulation by bacteria, viruses, or toxins, pattern recognition receptors (PRRs) on the cell surface (e.g., NLRP1, NLRP3, AIM2, or Pyrin) promote activation of downstream signaling pathways^29–32^. Upon recognizing these “danger signals,” these inflammasomes bind to the N-terminal PYD domain of the bridging protein apoptosis-associated speck-like protein containing a card (ASC). ASC then recruits and activates caspase-1 through caspase activation and the recruitment of domain (CARD)-CARD domain interactions^33^. In addition, a class of ASC-independent, CARD-containing inflammasomes (e.g., NLRC4) exists in cells. NLRC4 contains a CARD domain and directly binds and activates caspase-1 independently of ASC^34^. Activated caspase-1 specifically cleaves GSDMD, producing the GSDMD N-terminus and GSDMD C-terminus^21,35^. The GSDMD-N cleavage fragment is lipophilic and binds to the plasma membrane, disrupting its integrity^36^. Driven by osmotic pressure, water influx leads to cell swelling and membrane rupture, inducing pyroptosis. In addition, activated caspase-1 cleaves negatively charged interleukin (IL)-1β/18 precursors and promotes the production of positively charged mature IL-1β and IL-18, which are secreted extracellularly through GSDMD pores, where large negative charges accumulate, thereby causing an inflammatory response (**Figure 1B**)^16,18,19,37^.

The noncanonical pathway of pyroptosis is mainly mediated by caspase-4, caspase-5, and caspase-11. Upon stimulation of cells with LPS from Gram-negative bacteria, caspases-4/5/11 directly bind to and are activated by bacterial LPS^15,17^. Activated caspases-4/5/11 specifically cleave GSDMD, relieving the intramolecular inhibition of the GSDMD C-terminus on the GSDMD N-terminus, which leads to the formation of membrane pores. The formation of GSDMD pores disrupts ionic homeostasis. Although caspases-4, 5, and 11 are not directly involved in IL-1β and IL-18 production, the GSDMD pore causes K^+^ efflux, which activates NLRP3 inflammasomes, leading to caspase-1 activation^38,39^. Activated caspase-1 induces the secretion of IL-18 and IL-1β into the extracellular space, to amplify the inflammatory response. In addition, the formation of GSDMD pores is also accompanied by Ca^2+^ influx, which activates calpains and endosomal sorting complex required for transport (ESCRT) mechanisms. Calpains promote the rupture of pyroptotic cell membranes by degrading vimentin protein intermediate filaments^40^. However, the ESCRT mechanism repairs damaged membranes by contracting these membranes, thereby maintaining an “active state” while resisting cell death^41^. Based on this evidence, the transition from the “active” state to pyroptosis depends on the strength of the activation signal, the level of GSDMD expression and activation, and the activity of membrane repair mechanisms. In the caspase-11-mediated noncanonical pathway, Pannexin-1 is cleaved and releases intracellular ATP, which leads to P2X7-mediated pyroptosis^42^. Pannexin-1 cleavage also induces pyroptosis by activating NLRP3 inflammasomes and caspase-1^43,44^. In addition to caspases-1/4/5/11, the Ragulator-Rag complex is also involved in GSDMD membrane pore formation and inflammatory responses. Recent studies have reported that the Ragulator-Rag-mTORC1 signaling pathway leads to pore formation and pyroptosis by regulating GSDMD oligomerization, rather than membrane localization^45^.

In the past, caspases-3/8 were presumed to be apoptosis-associated caspases. However, various chemotherapeutic agents have recently been shown to mediate the cleavage of GSDME by inducing caspase-3 activation, leading to the pyroptosis of cancer cells^23,46^. Further studies revealed that *Yersinia pseudotuberculosis* inhibited TGF-β-activated kinase 1 (TAK1), and triggered the Rag-Ragulator complex, a critical factor involved in the progression of pyroptosis, which in turn caused mouse macrophage pyroptosis through the cleavage of GSDMD *via* the FADD-RIPK1-caspase-8 pathway^47–49^. These studies changed the previous misconception that *Yersinia* bacteria caused apoptosis in macrophages. Furthermore, it assigned a new function for apoptotic caspase-8 in the process of pyroptosis. During hypoxic conditions, TNF-α produced by macrophages induces the pyroptosis of cancer cells *via* the caspase-8-GSDMC pathway^50^. This mechanism explains the frequent formation of necrotic areas at hypoxic sites in tumor centers.

**Figure 2:**
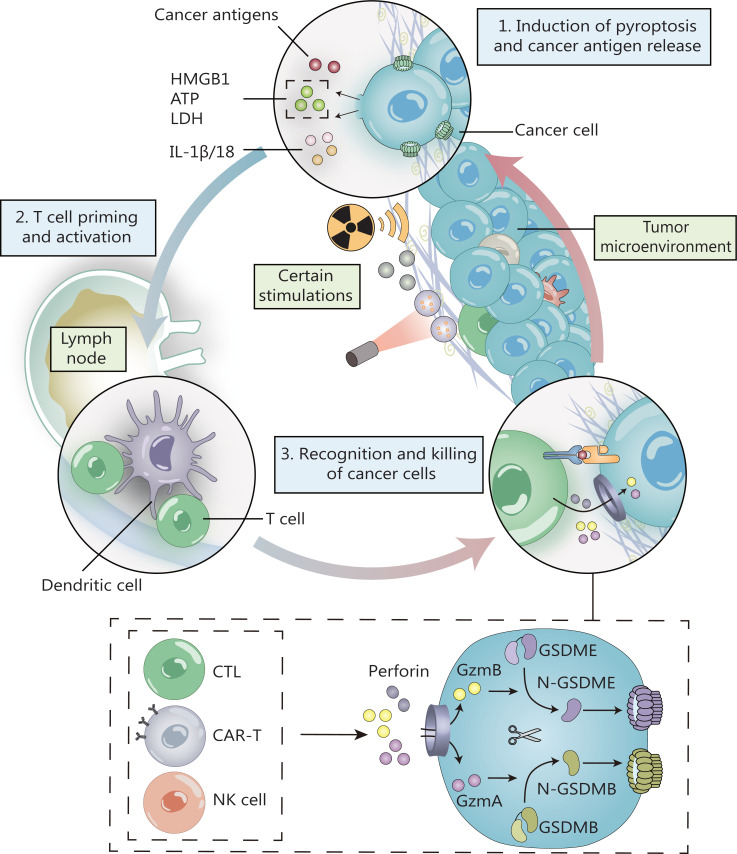
The positive feedback loop of pyroptosis and anti-tumor immunity. Pyroptotic cells release cancer antigens and large amounts of inflammatory substances. These antigens are presented by dendritic cells and induce activation of T cells. Activated NK cells and cytotoxic T lymphocytes secrete granzyme A (GzmA) and B (GzmB). GzmA directly cleaves GSDMB, while GzmB cleaves caspase-3 and GSDME. The cleaved GSDMB-N and GSDME-N fragments induce cancer cell pyroptosis and enhance antitumor immunity, creating a positive feedback loop of pyroptosis and anti-tumor immunity.

In addition, activated NK cells and cytotoxic T lymphocytes (CTLs) secrete granzyme A (GzmA) and B (GzmB). GzmA directly cleaves GSDMB, while GzmB cleaves caspase-3 and GSDME^51–53^. Activated GSDMB and GSDME induce the pyroptosis of cancer cells and enhance anti-tumor immunity, thus creating a positive feedback loop of pyroptosis and anti-tumor immunity (**Figure 2**). Based on these results, a small number of cancer cells undergoing pyroptosis mobilize a robust anti-tumor response to kill and clear tumors. These findings also further confirmed and refined the idea that the type of cell death is not determined by the type of caspase, but rather by the substrate recognized by caspases. Indeed, in the absence of GSDM, most of the proteases that mediate pyroptosis also induce apoptosis.

## The differences between pyroptosis, apoptosis, and necroptosis

**Figure 3:**
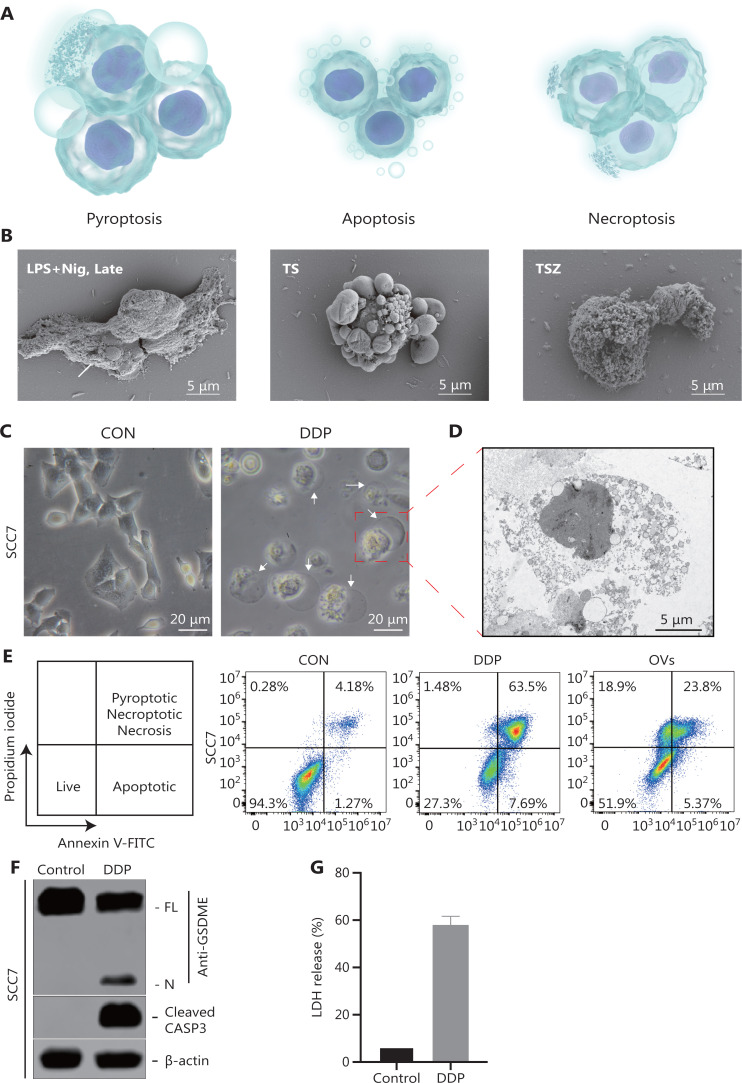
Representative results of pyroptosis detection. (A-B) Cellular morphological features during pyroptosis, apoptosis, and necroptosis. (A) Schematic diagram showing the morphology of cells undergoing pyroptosis, apoptosis, and necroptosis. (B) Scanning electron microscopy of SCC7 cells exposed to different treatments, to distinguish different forms of cell death. The arrow points to the bubbling of pyroptotic cells. (C) Representative microscopic images of SCC7 cells after cisplatin (DDP) treatment. White arrowheads indicate characteristic ballooning in cell membranes. (D) Representative transmission electron microscopy images of SCC7 cells treated with DDP. (E) Flow cytometry analysis of propidium iodide (PI)- and Annexin V-stained cells. SCC7 cells were treated with DDP and oncolytic viruses, respectively, for 24 h. Annexin V^−^/PI^−^ represents live cells, Annexin V^+^/PI^−^ represents apoptotic cells, and Annexin V^+^/PI^+^ denotes pyroptotic or necroptotic cells. (F) Western blot analysis of pyroptotic cell death markers in SCC7 cells treated with DDP. (G) Cytotoxicity of SCC7 measured by detecting lactate dehydrogenase release in culture supernatants. Nig, nigericin; TS, TNF + SMAC mimetic; TSZ, TNF + SMAC mimetic + caspase inhibitor z-VAD.

Pyroptosis, apoptosis, and necroptosis are genetically defined programmed cell death (PCD) pathways that have been widely studied. Various cell death modalities interact, and extensive crosstalk has been observed^54,55^, while different cell death modalities often have different mechanisms, are executed by different effector proteins, and exhibit different morphological features (**Figure 3 and Table 1**).

**Table 1:** The differences between pyroptosis, apoptosis, and necroptosis

	Pyroptosis	Apoptosis	Necroptosis
Character	Active PCD	Active PCD	Passive PCD
Inflammation	Yes	No	Yes
Morphology of cell membrane	Cell swelling, membrane rupture, bubble-like protrusions	Cell shrinkage, intact membrane, many vesicles of various sizes	Cell rounding and swelling, membrane rupture
Membrane blebbing	Yes	Yes	No
Membrane integrity	No	Yes	No
DNA damage	Yes	Yes	Yes
Chromatin condensation	Yes	Yes	No
Intact nucleus	Yes	No (fragmented)	Yes
Organelle morphology	Deformation	Intact	Swelling
Special constructions	Pyroptotic bodies	Apoptotic bodies	No
Release of intracellular contents	DAMPs, inflammatory molecules	No	DAMPs, inflammatory molecules
Associated molecules	Initiation: Caspase-1, 3, 4, 5, 11. Execution: GSDMD, GSDME	Initiation: Caspase-8, 9, 10. Execution: Caspase-3, 6, 7 Pro-apoptotic members: Bax, Bak, Bok. Anti-apoptotic members: Bcl-2, Bcl-xl, Mcl-1.	Initiation: RIPK1/RIPK3. Execution: MLKL. Inhibitory members: Caspase-8
7-AAD staining	Yes	No	Yes
EtBr staining	Yes	No	Yes
PI staining	Yes	No	Yes
PS exposure	Yes	Yes	Yes
Annexin V staining	Yes	Yes	Yes
TUNEL staining	Yes	Yes	Yes

PCD, programmed cell death; GSDM, gasdermin; EtBr, ethidium bromide; PI, propidium iodide; PS, phosphatidylserine; TUNEL, TdT-mediated dUTP nick end labeling.

For example, apoptosis and pyroptosis are characterized by caspase activation, DNA damage, and chromatin condensation, but the types of caspases involved and the associated cellular morphological features differ. As the first described PCD pathway, apoptosis is defined as a series of irreversible intracellular changes caused by activation of caspase family members by some pro-apoptotic stimuli such as endoplasmic reticulum stress and TNF-α. The main caspases involved in the apoptotic process include caspases-8/9/10, which are associated with apoptosis initiation, while caspases-3/6/7 are associated with apoptosis execution^56^. Caspases involved in the pyroptosis process mainly include caspases-1/3/4/5/8/11^9,56,57^. Morphologically, apoptosis is manifested as nuclear condensation, DNA damage, plasma membrane shrinkage, and blebbing, accompanied by formation of apoptotic bodies^56^. However, the cell membranes of apoptotic cells remain intact, so the cellular contents are not released. Unlike apoptosis, pyroptotic cells are characterized by the formation of many tiny pores in the cell membrane, which lead to the influx of water and swelling and expansion of cells, followed by rupture of cell membranes^9,25^. Pyroptotic cells show a significant release of cell contents with many bubble-like protrusions. In addition, DNA fragmentation in pyroptotic cells is random and causes a small degree of damage^25,58^. However, DNA fragmentation in apoptotic cells is ordered and accompanied by fragmentation of the nucleus^25,59^. In contrast to the “immunological silencing” of apoptosis, pyroptosis is accompanied by the development of a strong inflammatory response^60^.

In a similar manner, pyroptosis and necroptosis induce an inflammatory response, but the mechanisms mediating cell membrane rupture and the associated morphological features differ^61^. During necroptosis, cytosolic rupture is mediated by RIPK1, RIPK3, and MLKL^56,61^. However, during pyroptosis, cytosolic rupture is mediated by GSDM pores. Necroptosis is considered an alternate cell death mechanism to apoptosis and often occurs when apoptotic signaling components are inhibited. For example, caspase-8 is a “bridge” between apoptosis and necroptosis, resulting in the promotion of apoptosis. However, the necroptosis signal is antagonized by the caspase-8/cFLIP complex^62,63^. Because caspase-8 is also involved in the pyroptotic process, caspase-8 is considered a central regulatory molecule of all 3 cell death pathways^7^. In addition, necroptosis is morphologically manifested by rounding of cells and swelling of cells and organelles, which eventually lead to cytosolic rupture and cell death^61^. Unlike pyroptosis, necroptosis does not involve chromatin condensation and cell membrane blebbing, and the nucleus remains intact.

## Methods for monitoring pyroptosis

In recent years, as studies of pyroptosis have increased, new methods for monitoring cellular pyroptosis have emerged, and the previously used technics have become increasingly sophisticated. Based on morphological observations, techniques involving biochemistry, molecular biology, and immunology have been widely used to monitor pyroptosis (**Tables 2 and 3**).

**Table 2:** Methods for monitoring pyroptosis

	Indicators	Method	Reference
Changes in cell morphology	Cell swelling, membrane blebbing and rupture, bubble-like protrusions	Microscopy analysis	^23,27,48^
		TEM	^64,65^
		SEM	^21^
		Automated live cell imager	^66^
	GSDM-mediated pore formation	liposome leakage method	^67,68^
		AFM	^69,70^
Monitoring cell death	Cell viability	MTT/MTS assay	^71,72^
	DNA fragmentation	TUNEL method	^73,74^
Staining status	Annexin V/PI staining, SYTOX/7-ADD/EtBr/TO-PRO3 staining	Microscopy analysis, Flow cytometry	^23,25,53^
Molecular biomarkers	Cleavage of GSDM family (GSDMB/C/D/E)	Western blot Immunohistochemistry Immunofluorescence Q-PCR	^23,51,53,75,76^
	GSDM-Flag		
	Activation of Caspase-1/3/4/5/11		
	GzmA and GzmB		
	Released substances: IL-1β, IL-18, HMGB1, ATP, LDH	ELISA, ELISPOT	^27,72,77^
Other methods	The dynamic process of pyroptosis *in vivo*	Two-photon imaging technology	^78^

TEM, transmission electron microscopy; SEM, scanning electron microscope; GSDM, gasdermin; AFM, atomic force microscopy; GzmA, granzyme A; GzmB, granzyme B.

**Table 3:** Examples of methods for monitoring pyroptosis

Pyroptosis mechanism	Detection	Reference
Caspase-3/GSDME	Cell swelling with large bubbles	^23^
	Cleavage of GSDME	
	GSDME-mediated pore formation (liposome leakage method)	
	ATP cell viability	
	LDH release	
	PI/FITC staining	
GZMB/(Caspase-3)/GSDME	The changes in cell morphology	^53^
	Cleavage of GSDME	
	LDH, HMGB1 release	
	SYTOX green uptake	
	The use of zDEVD-fmk or zVAD-fmk to inhibit apoptosis and caspase-3-mediated pyroptosis	
GZMA/GSDMB	The changes in cell morphology	^51^
	Cleavage of GSDMB	
	Edman sequencing of the N termini to identify the cleavage sites	
	ATP–based cell viability LDH release	
	PI/FITC staining	
Hypoxia/TNF-α/GSDMC	Cell swelling with large bubbles	^50^
	Cleavage of GSDMC	
	Liposome leakage method	
	Cell death determined by LDH release	
	SYTOX green staining	

### Monitoring the changes in cell morphology

The most commonly used morphology-based detection techniques include light microscopy, fluorescence microscopy, confocal electron microscopy, scanning electron microscopy (SEM), transmission electron microscopy (TEM), and automated live cell imaging (**Figure 3B-D**). SEM distinguishes different forms of cell death based on changes in plasma membranes^21^. In addition, TEM indicates the location of various cellular structures with nanometer range resolution^64,65^. The entire cell death process can also be precisely observed and significantly distinguished from other cell death processes using automated live cell imagers^66^. For example, RAW-asc cells undergo pyroptosis after LPS + nigericin treatment^21^. Morphologically, pyroptotic cells swell and form bubble-like projections before the cells rupture. As the morphology changes, nuclear pyknosis and DNA strand breaks occur. When cells absorb water and rupture, many inflammatory cytokines and highly immunogenic molecules are released from dead cells, thus creating an “inflammatory” microenvironment. In addition, apoptosis occurs in TNF + SMAC mimetic-treated cells, as evidenced by the formation of apoptotic bodies^21^. Apoptotic RAW-asc cells have distinctly wrinkled membranes, but the cell membranes remain intact, and no release of contents is observed. Cells treated with TNF + SMAC mimetic + caspase inhibitor z-VAD (TSZ) undergo necroptosis^21^. Necroptotic RAW-asc cells swell and become round, resembling an overinflated balloon, and finally, the cell membrane ruptures, to release their contents.

Morphological detection of cellular pyroptosis is widely used because of its advantages of simplicity, intuitiveness, and unique morphological features. However, morphology-based techniques have certain limitations. For example, determination of the results is highly subjective and does not meet the requirements for quantitation. Therefore, morphology-based detection techniques are often used as a basis for other techniques.

Membrane pore formation has also been used to monitor the onset of pyroptosis. An assay based on liposome leakage methodology can be used *in vitro* to characterize the activity of GSDM^68^. Specifically, dipicolinic acid (DPA) is present outside liposomes loaded with terbium ions (Tb^3+^). The formation of GSDMD pores facilitates the leakage of Tb^3+^ and binding to external DPA to produce a robust fluorescent signal^68^. Hu et al.^67^ performed a screening using this method to identify disulfiram as an inhibitor of GSDMD pore formation. Visualization of pores based on the liposome leakage method is used to characterize GSDM activity, the effects of GSDM mutations, and other membrane proteins, indicating the plasticity and broad applicability of this method^67,68,79^. However, liposome experiments also have certain limitations. For example, the lack of membrane proteins and the underlying cytoskeleton leads to some possible discrepancies between the pores of liposomes and natural cell membranes.

Xia et al.^37^ reported the high resolution structure of the GSDMD pore and prepore using cryo-electron microscopy. They found a large amount of negative charges that accumulated within GSDMD pores, especially in the region close to the cytoplasm. Notably, the surface charge of pro-IL-1β is negative, while the surface charge of mature IL-1β is positive. Because both pro-IL-1β and IL-1β are much smaller than the pore size of GSDMD, the difficulty of pro-IL-1β passing through the GSDMD pore may be related to the physical principle of “homogeneous repulsion”^37^. This study overcame the stereotype that specificity of inflammatory factor release from GSDMD pores was based on the pore size. Furthermore, it elucidated the critical role of electrical charge in IL-1β release.

In contrast to cryo-electron microscopy, which provides data on 3-dimensional solids, atomic force microscopy (AFM) is limited to surface analysis. However, AFM images molecules in their native environment (e.g., in membranes). With high resolution and noninvasive imaging capabilities, AFM can characterize the membrane pore size and depth and can distinguish GSDM-forming pores from perforin- and complement-forming immune-associated pores^70^. Recent studies have analyzed the dynamics of GSDMD pore formation using high resolution AFM (≤ 2 nm)^35,69^. Mulvihill et al.^69^ found that GSDMD-N bound to lipid membranes and oligomerized into slit- and arc-shaped structures, which in turn fused to form a ring-like transmembrane pore structure. The visualization of these structures provides the basis for developing drugs based on GSDM, and should further increase our understanding of GSDM-mediated pyroptosis.

### Monitoring staining status

Reliance on changes in cell morphology is insufficient to determine the occurrence of pyroptosis. Cell proliferation and survival analyses using MTT and MTS methods are often used as auxiliary methods to cell morphological observations, to confirm the occurrence of pyroptosis^71,72^. In addition, cells can be prestained with propidium iodide (PI) or SYTOX to aid in the overall detection process when using a microscope to observe the morphology of the cell death process^27,71,80^.

Annexin V/PI is commonly used in fluorescence microscopy and flow cytometry to detect cell death. It shows different staining patterns depending on the type of cell death^23,77,81^. In normal cells, Annexin V and PI do not penetrate cell membranes, so the cells are not stained (**Figure 3E**). In addition, the integrity of the cell membrane is not affected during apoptosis, so PI cannot penetrate cell membranes to stain intracellular DNA^23,81^. However, phosphatidylserine (PS) flips from the inner side of the cell membrane to the outer side of the cell membrane, and is recognized by Annexin V. Furthermore, PS externalization during apoptosis promotes phagocytic clearance in apoptotic cells to avoid inflammation. Thus, flow cytometry shows positive Annexin V staining in apoptotic cells. When cells undergo pyroptosis, PI freely enters the cell to label DNA, due to the increased permeability of cell membranes, so PS on the inner side of the cell membrane is labeled by Annexin V. Pyroptotic cells will therefore appear to change directly from PI^−^ and Annexin V^−^ staining to PI^+^ and Annexin V^+^ staining^23,81^. Notably, when apoptotic cells are not cleared *in vivo* in a timely manner, the cells undergo secondary necrosis, and cell membranes rupture. PI penetrates cell membranes to stain intracellular DNA, and the cells change from single Annexin V^+^ staining to PI^+^ and Annexin V^+^ staining^82^. Flow cytometry and Annexin V/PI staining therefore allow researchers to monitor and determine, in real time, whether cells undergone pyroptosis.

SYTOX green is a green nucleic acid dye that readily passes through the plasma membranes of damaged cells, but not through the plasma membranes of living cells. Compared with PI staining, SYTOX green has a higher affinity for nucleic acids, and its fluorescence intensity is increased more than 500-fold upon binding to nucleic acids^55,67^. Therefore, the onset of cell pyroptosis can be more sensitively determined based on the uptake of SYTOX green by cells. In addition, some other low molecular weight dyes such as 7-amino actinomycin (7-AAD) and ethidium bromide (EtBr), stain both pyroptotic and necroptotic cells, and thus distinguish them from apoptotic cells^83,84^.

### Monitoring molecular biomarkers for pyroptosis

Molecular biomarkers used for pyroptosis detection include intracellular markers (such as caspases1/3/4/5/8/11, granzyme, and the GSDM family), and cell-released substances [HMGB1, ATP, IL-1β/18, lactate dehydrogenase (LDH), and reactive oxygen species (ROS)]^9,85^. Currently, with the development of various technologies based on biochemical characteristics (such as flow cytometry), researchers can more accurately detect and characterize pyroptosis.

The pyroptosis signaling pathway is blocked in normal cells, and the GSDM family, IL-1β/IL-18, and caspases are not activated^9,24^. Pyroptosis is activated by caspases or granzyme, accompanied by cleavage of the GSDM family and release of IL-1β/IL-18^9^. Consistent with the requirements of investigators, the related markers can be used to monitor pyroptosis by: (1) monitoring the cleavage of GSDMB/C/D/E, (2) monitoring the activation of caspases-1/3/4/5/8/11 and GzmA/GzmB to determine the signal transduction pathway, and (3) monitoring the release of pyroptosis substances. Because the regulation of pyroptosis rarely involves transcription and translation, immunological techniques such as Western blot (WB) have become the gold standard for detecting pyroptosis (**Figure 3F**). Based on protein markers of pyroptosis, almost all studies have used WB to verify the role of pyroptosis, especially the detection of GSDM processing and caspase activation and expression^23,51,53,75,76^. A FLAG tag can also be used to detect the distribution of GSDMs in cells.

ELISAs or ELISPOT assays are also often used to detect the release of various substances, such as IL-1β, IL-18, and HMGB1, which will be released outside the cell upon rupture of cell membranes^27,72,77^. LDH is expressed in the cytoplasm as an indicator of cell death. During pyroptosis, LDH is released from cells when cell membrane permeability is altered, and in turn, is detected in the supernatant (**Figure 3G**)^23,27,77^. Furthermore, Ca^2+^ influx has also been shown to be related to the occurrence of pyroptosis. Some studies have reported that Ca^2+^ is a prerequisite for functioning of GSDMD-N. Ca^2+^ influx activates NLRP3 inflammasomes, thus triggering GSDMD-mediated cell pyroptosis^86,87^. The detection of Ca^2+^ can therefore help to confirm the occurrence of pyroptosis. For example, Zhao et al.^88^ incubated 4T1 cells with pyroptosis-inducing photosensitive materials followed by addition of the Ca^2+^ fluorescent probe, Fluo-8AM, then stained them with the fluorescent dye, Hoechst 33258. The intracellular Ca^2+^ concentration was analyzed qualitatively or quantitatively by observing and detecting the fluorescence intensity after treatment using confocal microscopy or flow cytometry, respectively.

### Monitoring pyroptosis *in vivo*

Compared with the previously mentioned *in vitro* detection methods, *in vivo* detection methods for pyroptosis are relatively simple. In the past, immunohistochemical methods were often used to detect *in situ* cell death in different organs, such as the commonly used TdT-mediated dUTP nick end labeling (TUNEL) method to detect cell death^73,74^. Now, the TUNEL method can be combined with molecular biomarkers related to pyroptosis to monitor pyroptosis. This process includes immunohistochemistry and immunofluorescence staining for the upstream pathway of pyroptosis-specific proteases (caspases-1/3/4/5/8/11), GzmA, GzmB, and the GSDM pyroptosis-executing proteins^89^. In addition, better detection of pyroptosis is possible by analyzing soluble extracellular biomarkers such as LDH released by dead cells in the plasma, combined with the detection of pyroptosis-related molecular biomarkers^90^.

Because the current immunohistochemical method for GSDMs at the tissue level has difficulty distinguishing the entire length of GSDMs from cleaved GSDM fragments, the most commonly used method for detecting pyroptosis activity *in vivo* is WB, which detects changes in the protein levels of full-length GSDM and GSDM fragments in cells, and detects the hydrolytic activation of GSDMs by changes in size. In addition, WB has also been used to monitor the levels of caspase family members (caspases-1/3/4/5/8/11), GzmA, and GzmB, as well as HMGB1, IL-1β, IL-18, and other released substances^27,51,53,89,91^.

## Conclusions

Pyroptosis is a regulated form of cell death that depends on the formation of plasma membrane pores by GSDM family proteins and is often, but not always, accompanied by activation of inflammatory caspases^56,92^. In the presence of PAMPs or cell-derived DAMPs, different caspases or granzymes mediate cleavage of GSDM family members, subsequently causing altered membrane permeability, cell swelling, and membrane rupture^56^. Cell pyroptosis is a “double-edged sword.” Using this process, multiple strategies to induce pyroptosis in cancer cells have been developed for cancer therapy. Tumors resistant to ICIs are considered “cold tumors” and exhibit a lack of T cell infiltration^93,94^. Induction of pyroptosis can transform “cold” tumors lacking infiltrating T cells into “hot” tumors containing high levels of infiltrating T cells, thereby improving the efficacy of ICIs. However, pyroptosis can also cause damage to the body. For example, SARS-CoV-2 infection induces inflammatory cytokine storms by causing cell pyroptosis to release a large amount of inflammatory substances^95^. In addition, based on the theory of inflammation-cancer transformation and chronic inflammation-induced carcinogenesis, pyroptosis, a proinflammatory death pathway, can form a microenvironment suitable for cancer cell growth^26,96^. As a critical factor involved in inflammation, pyroptosis mediated by GSDMs plays an essential role in treating many diseases. The development of effective pyroptosis agonists or antagonists is expected to improve the treatment of some inflammatory diseases and tumors^47,48,67,94,97^. In the future, there should be extensive efforts to identify drugs that modulate pyroptosis. Monitoring the occurrence of pyroptosis is therefore very important, and will directly affect the process used to screen pyroptosis inducers and inhibitors.

**Figure 4:**
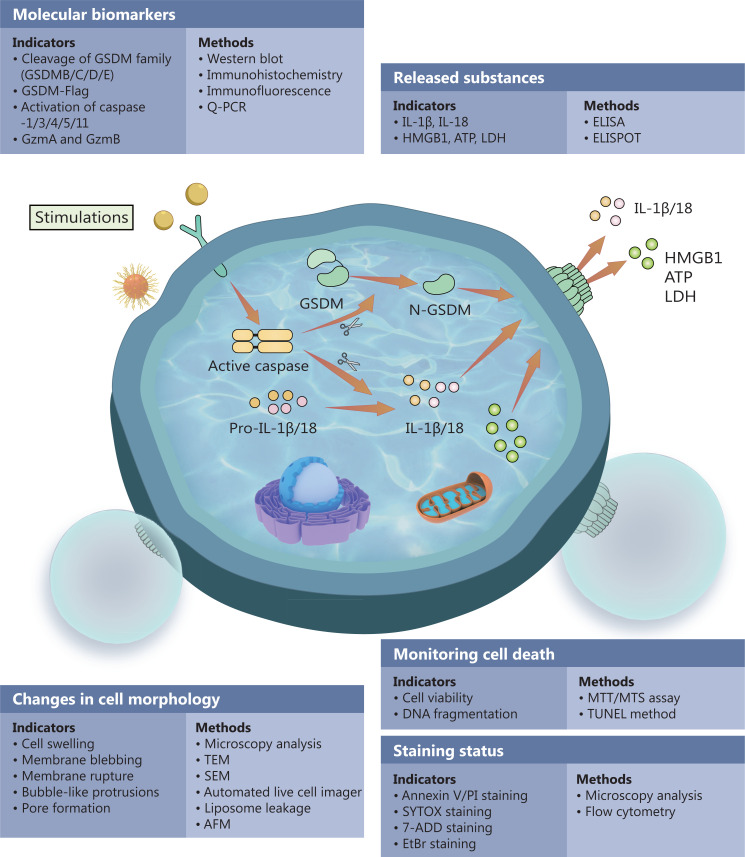
Methods for monitoring pyroptosis. Pyroptosis is a multistep process. In practical applications, a combination of assays is often used to monitor the occurrence of pyroptosis and accurately determine pyroptosis results of experimental cells. GSDM, gasdermin; GzmA, granzyme A; GzmB, granzyme B; TEM, transmission electron microscopy; SEM, scanning electron microscope; AFM, atomic force microscopy.

However, to date, systematic descriptions of the detection of pyroptosis are lacking. In this review, we described the current standard *in vivo* and *in vitro* pyroptosis detection methods (**Figure 4**). Notably, cell pyroptosis is a complex process. In practice, a combination of assays is often used to monitor the occurrence of pyroptosis to accurately determine pyroptosis results in experimental cells, and further analyze the in-depth results to draw corresponding conclusions^98^. Furthermore, a combination of pyroptosis-related markers can be used to detect pyroptosis. These markers include cysteine aspartate-specific protease (caspases-1/3/4/5/8/11) activation, GzmA and GzmB activation, cleavage-mediated activation of GSDMs, and the release of LDH, HMGB1, IL-1β, IL-18, and other substances.

At present, there are many challenges in detecting pyroptosis, especially at the cellular level *in vivo*. In addition, how to detect ion channels formed during cell pyroptosis, and how to best characterize the complex ESCRT process are also questions that need to be addressed. New methods must therefore be developed to study the process of pyroptosis. Pyroptosis is a rapid mode of death accompanied by phagocytosis and clearance by macrophages^9,25^, so it is difficult to detect the timing and location of pyroptosis *in vivo*. In the future, we could genetically encode the activity reporter gene of the pyroptosis-related protein and introduce it into target cells that we want to detect, and then use 2-photon imaging technology to identify the cells that undergo pyroptosis. Using this technology to introduce the gene-encoded caspase-3 activity reporter gene into primitive T cells, to characterize T cells that are about to undergo apoptosis *in vivo* has been reported^78^. In addition, some researchers have made transgenic and knock-in mice expressing apoptosis reporter genes and observed dying B cells using the fluorescence resonance energy transfer indicator of apoptosis reporter, combined with intravital 2-photon microscopy^99,100^. These reports suggest that we can use the same technique to observe cells *in vivo* that undergo pyroptosis. Based on the feature that pyroptosis is triggered by the accumulation of the N-terminus of the pyroptosis protein in cell membranes, we can fluorescently label the N-terminus of the pyroptosis protein. This method can determine whether the cell is about to undergo pyroptosis, as well as the degree of pyroptosis, based on whether the N-terminus of the pyroptosis executive protein aggregates on the cell membrane, as well as on the number of aggregates. Targeting mitochondria is a new intervention strategy to regulate cell death. A close relationship exists between pyroptosis and mitochondrial apoptosis^101^. The use of quantum dot fluorescence to detect changes in the mitochondrial transmembrane potential and permeability during mitochondrial apoptosis should also contribute to the study of pyroptosis.

By using high resolution techniques such as cryo-electron microscopy and AFM in the analyses of critical molecular structures of cell pyroptosis, researchers have gained a better understanding of the scorch death/pyroptosis mechanism^37,69^. In future studies, it may be necessary to define a pyroptosis index modeled on the apoptosis index, which will help evaluate the effects of new anti-tumor methods. With the rapid development of technologies and the joint applications of multidisciplinary approaches (e.g., chemistry, materials science, and life science), additional methods for detecting cell pyroptosis with high accuracy, high specificity, and low time and cost will likely be developed.
